# Intrauterine Adhesions following Conservative Treatment of Uterine Fibroids

**DOI:** 10.1155/2012/853269

**Published:** 2011-11-28

**Authors:** Pietro Gambadauro, Johannes Gudmundsson, Rafael Torrejón

**Affiliations:** ^1^Centre for Reproduction, Department of Obstetrics and Gynaecology, Uppsala University Hospital, 751 85 Uppsala, Sweden; ^2^Department of Obstetrics, Gynaecology and Breast Pathology, Virgen del Rocío University Hospital, University of Seville, 41013 Seville, Spain

## Abstract

Uterine fibroids are common in women of reproductive age and various conservative treatments are available. In order to achieve a successful conservative treatment of fibroids, functional integrity of the uterus is as important as tumor removal or symptoms relief. In this context, intrauterine adhesions must be recognized as a possible complication of conservative management of uterine fibroids, but diagnostic pitfalls might justify an underestimation of their incidence. Hysteroscopic myomectomy can cause adhesions as a result of surgical trauma to the endometrium. The average reported incidence is around 10% at second-look hysteroscopy, but it is higher in certain conditions, such as the case of multiple, apposing fibroids. Transmural myomectomies also have the potential for adhesion, especially when combined with uterine ischemia. Uterine arteries embolization also carries a risk of intracavitary adhesions. Prevention strategies including bipolar resection, barrier gel or postoperative estradiol, might be useful, but stronger evidence is needed. In view of current knowledge, we would recommend a prevention strategy based on a combination of surgical trauma minimization and identification of high-risk cases. Early hysteroscopic diagnosis and lysis possibly represents the best means of secondary prevention and treatment of postoperative intrauterine adhesions.

## 1. Introduction

Uterine fibroids are an extremely common finding in women of reproductive age, and various conservative treatment approaches are available.

Indications to conservative treatment might be represented by the patient's wish to avoid a hysterectomy or to preserve or enhance her reproductive potential.

In the latter case, functional integrity of the uterus is as important as the complete removal of the fibroid tumors or symptoms relief, in terms of surgical outcomes and success.

Women, undergoing major gynaecological surgery, have a high risk of developing postoperative adhesions of some extent [1]. This circumstance, although commonly considered inevitable, represents a short-/long-term complication of surgery, with important repercussions on patients' health and quality of life, as well as relevant direct and indirect costs for the healthcare systems [2].

Adhesions in gynecology have a particular relevance, because of the potential impact on reproductive function, on top of the known consequences, such as abdominal/pelvic pain or bowel obstruction. Therefore, medical literature of the last decades has dedicated great attention to the topic of adhesion prevention after “gynaecological surgery” by focusing on peritoneal adhesion, but not on intracavitary adhesions [3]. Nevertheless, intrauterine adhesions are a possible complication of therapeutic procedures on the uterus and, although often silent, can interfere with fertility and always hide the potential of becoming symptomatic, for example, the Asherman's syndrome.

This paper focuses on intrauterine adhesions that might occur as a result of conservative management of uterine fibroids.

## 2. Intrauterine Adhesions following Treatment of Submucous Fibroids

Hysteroscopic myomectomy is currently the gold standard for the surgical treatment of submucosal fibroids, having replaced traditional surgical approaches such as hysterectomy and abdominal myomectomy. It was first described in 1976 by Neuwirth and Amin, who used an urologic resectoscope [4], while the first report of a gynaecological instrument came by Hallez in 1987 [5]. Resectoscopic myomectomy is safe and effective in removing fibroids and treating related symptoms [6], and a wide range of instruments is now available [7].

As any other intrauterine operation, hysteroscopic myomectomy can cause adhesions as a result of surgical trauma to the endometrium. Hysteroscopic surgery is commonly considered as a minor risk when compared with the interventions with the highest adhesiogenic potential, such as dilatation and curettage (D and C) following delivery or miscarriage [8]. Nevertheless, pitfalls in the diagnosis of postoperative intrauterine adhesions might cause an underestimation of the problem, and a second-look hysteroscopy would be needed to calculate the real incidence (Table 1). 

In a prospective study by Taskin et al., a second-look diagnostic hysteroscopy showed mild intrauterine adhesions in the 37.5% of patients after monopolar resection of a single fibroid, and in the 45% after resection of multiple fibroids [9]. Interestingly, a lower incidence of adhesions was reported by the same study following the resection of polyps (3.6%) or uterine septa (6.5%), and no differences were found between patients who were pretreated with danazol and untreated ones. The incidence of adhesions reported by Taskin et al. is definitely high but could be justified by the short interval between primary surgery and hysteroscopic followup. As a matter of fact, the latter was conducted between 14 and 30 days after the fibroid resection, and the same Authors reported doubts whether the adhesions were “de novo”, or part of the normal healing process.

In contrast with those findings, Yang et al. report a low rate of 1.5% of adhesions at 1-to-3-month hysteroscopic second look following the removal of a single submucous fibroid, while, in their experience, adhesion rate after resection of apposing fibroids reaches the 78%, in spite of the insertion of an intrauterine device (IUD) postoperatively [10]. Interestingly, a subgroup of seven patients, who were operated for multiple apposing fibroids and did not receive an IUD, underwent an early lysis of adhesions at 1-2 weeks from the primary surgery, and none of them presented with adhesions at the scheduled 1–3-month second look.

In the setting of a larger, randomized study on the prevention of adhesions with auto-cross-linked hyaluronic acid gel following resectoscopic surgery, Guida et al. diagnosed postoperative adhesions in one fourth of patients submitted to fibroid resection [11]. However, the rate of adhesions, detected at a 3-month hysteroscopic second look, was significantly lower when auto-crossed hyaluronic acid gel was used following fibroid resection (16% cases versus 33.33% controls), although larger, and adequately powered, trials would be needed to confirm this finding. In this study, the fibroid resections were accomplished with bipolar resectoscopes. These instruments are currently replacing the older generation of monopolar instruments because of the invaluable advantage of using an electrolyte-containing isotonic distension medium such as normal saline. The reduction of the risks of electrolyte imbalance related to fluid overload [12] increases the safety profile of this kind of surgery.

A role of bipolar resectoscopes in reducing the risk of postoperative intrauterine adhesions has been suggested by Touboul et al. [13]. These Authors reported the findings of systematic second-look hysteroscopy following bipolar hysteroscopic myomectomy, demonstrating synechiae only in 4 out of a group of 53 infertile patients (7.5%). The latter evidence is anyway weak and not supported by comparative studies. Moreover, low rates of intrauterine adhesions have also been reported following monopolar resection of fibroids.

Roy et al., for instance, retrospectively analyzed the two-month second-look hysteroscopy in 186 patients with infertility and recurrent abortions submitted to myomectomy with monopolar resectoscope, showing adhesions in only 2 patients (1.07%) [14]. However, all the patients in this study had received intra and postoperative antibiotic prophylaxis, as well as a course of estradiol valerate, 2 mg per day, during 30 days.

Finally, in a series of five patients with diffuse uterine leiomiomatosis who underwent selective hysteroscopic resection, published by Yen et al. in 2007, postoperative intracavitary adhesions were found in 2 cases [15]. Interestingly, one out of those two patients had developed hypomenorrhea and had repeat hysteroscopic adhesiolysis, but also conceived spontaneously at 4 months following the last surgery, and eventually delivered an healthy infant (cesarean section for breech presentation) after an uneventful pregnancy.

## 3. Intrauterine Adhesions following Treatment of Intramural Fibroids

The potential role of hysteroscopic fibroid surgery in inducing intrauterine synechiae is obvious. Nonetheless, also other conservative treatments of uterine fibroids might lead to intracavitary adhesions.

Myomectomy, both abdominally and laparoscopically, is a common and safe conservative surgical procedure for intramural fibroids, especially in women of reproductive age [16]. The medical literature proves its role in symptom relief and fertility preservation, although it is still debated as a purely fertility-enhancing procedure in infertile patients.

The occurrence of abdominal and pelvic adhesions as a complication of open or laparoscopic fibroid enucleation is well documented [17]. On the contrary, intrauterine synechiae are not commonly addressed as a potential risk of myomectomy. Indeed, reasonable evidence exists on the development of adhesions following transmural surgery, such as caesarean sections or abdominal myomectomy [18, 19].

The overall risk following myomectomy is considered low (1.3%) [20], but the heterogeneity of this kind of surgery (e.g., not all the fibroids are transmural, and not all the abdominal myomectomies require the opening of the endometrial cavity) makes it difficult to study the association between myomectomies and risk of synechiae.

Moreover, synechiae can be seen at hysterosalpingography [18] and hysteroscopy [19], but those diagnostic procedures are not routinely used postoperatively. In addition, we need to be aware of certain groups of patients, or procedures, that might increase the risk of adhesions.

Several approaches have been studied and proposed to facilitate myomectomy or reduce feared complications such as hemorrhage, and the related risk for hysterectomy, although their potential effect on the uterine cavity has seldom been assessed. Tixier et al. studied the effect of preoperative uterine arteries embolization (UAE) and uterine arteries surgical ligation on the outcomes of laparoscopic or open myomectomy [21]. The patients wishing to conceive after myomectomy were submitted to diagnostic hysteroscopy 3 months after surgery. The authors reported an incidence of 18% (4/22) of synechiae in women whose myomectomy had been preceded by a temporary uterine artery embolization. On the contrary, no intrauterine adhesions were found among the cases where the uterine arteries were ligated intraoperatively by mono- or bilateral reabsorbable clips. The same measure was 14.8% (4/27) for patients who had not received any preparation prior myomectomy.

The same research group also reported on retrospective findings of hysteroscopic evaluation 3 months following myomectomy with previous UAE, in patients wishing to conceive [22]. In that case, three out of the ten patients presented intrauterine synechiae (30%). 

Uterine artery embolization under X-ray guidance is also the main nonsurgical alternative to myomectomy [23]. It was initially described in 1995 [24], and it is an effective treatment in reducing symptoms such as bleeding or pelvic pain and also induces shrinkage of the tumors [20]. It is contraindicated in case of intracavitary fibroids, because of a risk of spontaneous expulsion [25]. UAE is controversial for fertility wishing patients since long-term effects on ovarian function and fertility are not known, and complicated obstetrical outcomes have been reported [26, 27].

In a study by Mara et al. on women of fertile age undergoing UAE for symptomatic uterine fibroids, hysteroscopy performed at 3 to 9 months from the embolization showed a high prevalence of pathological or abnormal findings, among which 14% of intrauterine or cervical adhesions (7 out of 51 patients) [28]. These findings demonstrate that surgical trauma is not essential for the development of synechiae and support the doubts existing on the suitability of UAE for fertility wishing patients, in spite of the evidence of successful pregnancies published in recent years [29].

## 4. Discussion

While the mechanism of adhesion formation is still largely unknown, and multiple predisposing and causal factors are probably implicated, trauma to the endometrium is commonly considered the major factor in the genesis of uterine synechiae.

The endometrium is composed of two layers, a functional layer and an underlying basal layer. The latter is needed for regenerating the functional layer, which is lost with the menstruation. Trauma to the basal layer can lead to the development of intrauterine scars resulting in adhesions, which can obliterate the cavity to varying degrees. A peculiarity of intrauterine trauma is that it often occurs simultaneously on apposing surfaces, because of the limited volume of the cavity. This is quite evident in case of blind procedures, such as a dilatation and curettage. The association between trauma, synechiae, and specific symptoms is what had already been identified by Joseph Asherman in the first half of last century (*amenorrhoea traumatica*) [30].

As a consequence of trauma, a tissue healing process is started, and it can progress by two different modalities: regeneration or repair. Regeneration occurs cyclically after the menstruation, when the lost tissue is replaced by a new functional layer, originating by a healthy basal layer. A repair mechanism, instead, replaces the missing normal tissue with an extracellular matrix (e.g., fibronectin and collagen), leading to scar formation. As such scarring could be considered a failure of tissue regeneration.

Postsurgical adhesions develop in a similar fashion as scars, that is, within the repair healing process. Initially, the injury is covered and sealed by fibrin (filmy, “fibrinous” adhesions). Most commonly, physiologic fibrinolysis is able to limit the extent of those filmy adhesions and dissolve them. Factors such as a persistent or extended tissue trauma might disrupt the process of fibrinolysis. When that occurs, collagen and other matrix substances are produced by repair cells such as fibroblasts or macrophages, resulting into permanent fibrous adhesions [31].

Tissue hypoxia is thought to be a factor that potentiates the initial tissue injury and triggers a cascade of responses that leads to the creation of adhesions [32, 33]. Hypoxia negatively affects fibrinolysis [34], and in vitro studies demonstrate that it also induces irreversible phenotypic changes in fibroblasts [35]. 

The current knowledge of the mechanism of adhesion formation is certainly not exhaustive but justifies the clinical findings of higher rate of synechiae following removal of multiple, apposing fibroids (extended trauma) or UAE (hypoxia).

Nevertheless, some patients develop adhesions regardless of the extent of the trauma or other plausible risk factors. Moreover, the diagnosis of silent intrauterine adhesions is not straightforward, and we believe that their incidence might be underestimated. As a matter of fact, the main diagnostic tool used in gynaecology, ultrasonography, does not seem to be accurate in diagnosing synechiae, and hysteroscopy should be considered the gold standard. For instance, systematic pre-IVF outpatient hysteroscopy in patients with normal findings at HSG shows 4.1% of adhesions, whereas ultrasound could not detect any [36]. Moreover, hysteroscopy identifies intrauterine adhesions in 11% of patients with repeated failure of IVF-ET, none of them suspected at standard TV ultrasound [37]. It is still a matter of debate whether infertile patients should undergo systematically a diagnostic hysteroscopy [38], but we believe that those at a higher risk of synechiae, such as following a multiple resection of fibroids, should be offered an endoscopic assessment of their uterine cavity, which is a method with high compliance, that can be performed in an outpatient setting without any need for anesthesia [39].

Prevention of synechiae has not been exhaustively studied in medical literature. Proposed strategies mostly focus on etiopathology. For instance, IUDs have been advocated, in order to avoid apposing surfaces postoperatively but have not been proven effective [10]. Some authors have also proposed intrauterine balloons, such as foley catheters, but the benefits of these intrauterine devices are not clearly higher than, for instance, the risk of postoperative infections [40].

Reabsorbable barriers such as auto-cross-linked hyaluronic acid gel have been shown to significantly reduce adhesions' reformation and severity after hysteroscopic adhesiolysis [41] and might be effective after resectoscopic myomectomy because of high sensitivity and prolonged intracavitary residency time [11, 40].

Postoperative treatment with oral estrogens has been used, in order to stimulate endometrial regeneration [14]. Although the potential effect of an estrogenic stimulus on the endometrium appears logical, available evidence supporting its use is not strong, and, therefore, they cannot be recommended routinely. On the contrary, it seems reasonable to avoid, when possible, any iatrogenic hypoestrogenic status, such as that induced by preoperative GnRH agonists, whose role in facilitating surgery has been suggested but is still controversial [3, 42, 43].

Finally, although it has been proposed that infection might cause adhesions, no evidence supports the prophylactic use of antibiotics for primary hysteroscopic surgery or synechiolysis [40, 44].

Surgical strategies might also offer ways to prevent synechiae. For instance, resection of apposing fibroids could be avoided, by the adoption of two-step procedures. Minimizing tissue trauma by reducing thermal injury and preferring mechanical instruments is possible during resectoscopic myomectomy [45]. The use of bipolar resectoscopes is recommendable because of overall advantages, but we lack comparative studies to prove their superiority on monopolar counterparts in terms of postoperative synechiae. Reducing the size of the instruments could also potentially play a role, but it is limited by the volume of the fibroids [46, 47].

In case of myomectomies for intramural fibroids, intraoperative techniques to reduce bleeding, such as those by endoscopic loops or ligations [48–50], might be preferable over preoperative UAE [22]. Identification and separate suturing of different layers, especially in case of opening of the endometrial cavity, is recommendable.

Finally, performing a second-look or control hysteroscopy as a followup of the primary surgery, especially in high risk cases, seems to be a feasible and effective way to diagnose and treat synechiae, often at their early, fibrinous stage [10].

## 5. Conclusions

The conservative treatment of fibroids on women of reproductive age is also necessarily a functional treatment. Both the anatomy and the functionality of the uterus need to be respected, preserved, and in some cases improved. In this context, postoperative intrauterine synechiae, although considered uncommon, must be considered as potentially serious complications of fibroids' treatment.

Hysteroscopic resection of fibroids might cause synechiae, especially in the case of multiple, apposing fibroids. Transmural myomectomies also have adhesiogenic potential, especially when combined with uterine ischemia. Uterine arteries embolization cannot be considered a first choice for patients with fibroids who wish to conceive, also because it carries a risk of intracavitary adhesions.

The real occurrence of intrauterine adhesions following myomectomy might be underestimated because of diagnostic pitfalls and low awareness [51].

Various strategies have been suggested for the prevention of postoperative uterine synechiae, but we are lacking well-powered and designed studies to assess their value after myomectomy or, for instance, UAE.

In view of current knowledge, we recommend a prevention strategy based on a combination of good surgical practice and awareness of high-risk cases and procedures. 

Surgery should minimize the damage to healthy tissue and avoid simultaneous trauma on apposing endometrial surfaces. Identification of high-risk patients, followed by early hysteroscopic diagnosis and lysis of postsurgical synechiae, possibly represents the best means of secondary prevention and treatment of intrauterine adhesions. 

## Figures and Tables

**Table 1:** Intrauterine synechiae following resectoscopic myomectomy. Second-look hysteroscopy.

Author	Instrument	Second-look interval	Fibroids	Additional treatment	Adhesions/second-look hysteroscopies	Adhesion rate
Taskin et al. 2000 [9]	monopolar	14–30 days	single	no/placebo	8/22	36.36%
				Danazol	7/20	35%
			multiple	no/placebo	6/13	46.15%
				Danazol	6/14	42.85%

Guida et al. 2004 [11]	bipolar	3 months	single*	no/placebo	8/24	33.33%
				a–c hyaluronic acid gel	4/25	16%

Yang et al. 2008 [10]	monopolar*	1–3 months	single	No	2/132	1.5%
			2, nonapposing	IUD, 1 month	0/5	0%
			≥2 apposing	IUD, 1 month	7/9	78%
			≥2 apposing	early lysis 1-2 weeks	0/7	0%

Touboul et al. 2009 [13]	bipolar	2 months	single*	No	4/53	7.5%

Roy et al. 2010 [14]	monopolar	6 weeks	single	estradiol valerate (6 weeks) antibiotics (5 days)	2/186	1.07%

Total					54/510	10.58%

*Extrapolated, but not clearly stated on the original paper.
